# Supplementary material for: Dihydroartemisinin imposes positive and negative regulation on Treg and plasma cells via direct interaction and activation of c-Fos
Source: Commun Biol. 2023 Jan 16;6:52. doi: 10.1038/s42003-023-04454-5 (PMC9842609; doi:10.1038/s42003-023-04454-5)
Supplement: Supplementary file 2 — Supplementary Information [file 42003_2023_4454_MOESM2_ESM.pdf]

Supplementary Information

Dihydroartemisinin imposes positive and negative regulation on T_{reg} and plasma cells via direct interaction and activation of c-Fos

Authors: Qilong Li[1,2#], Ning Jiang[1,2#], Yiwei Zhang[1,2], Yize Liu[1,2], Ziwei Su[1,2], Quan Yuan[1,2], Xiaoyu Sang[1,2], Ran Chen[1,2], Ying Feng[1,2], Qijun Chen[1,2*]

Affiliations

[1]Key Laboratory of Livestock Infectious Diseases, Ministry of Education, Key Laboratory of Zoonosis, College of Animal Science and Veterinary Medicine, Shenyang Agricultural University, 120 Dongling Road, Shenyang 110866, China

[2]Research Unit for Pathogenic Mechanisms of Zoonotic Parasites, Chinese Academy of Medical Sciences, 120 Dongling Road, Shenyang 110866, China

[#] These authors contributed equally.

[*] Corresponding author. Email: qijunchen759@syau.edu.cn.

Table of Contents:

Supplementary Figures: 7

Supplementary Data: 3

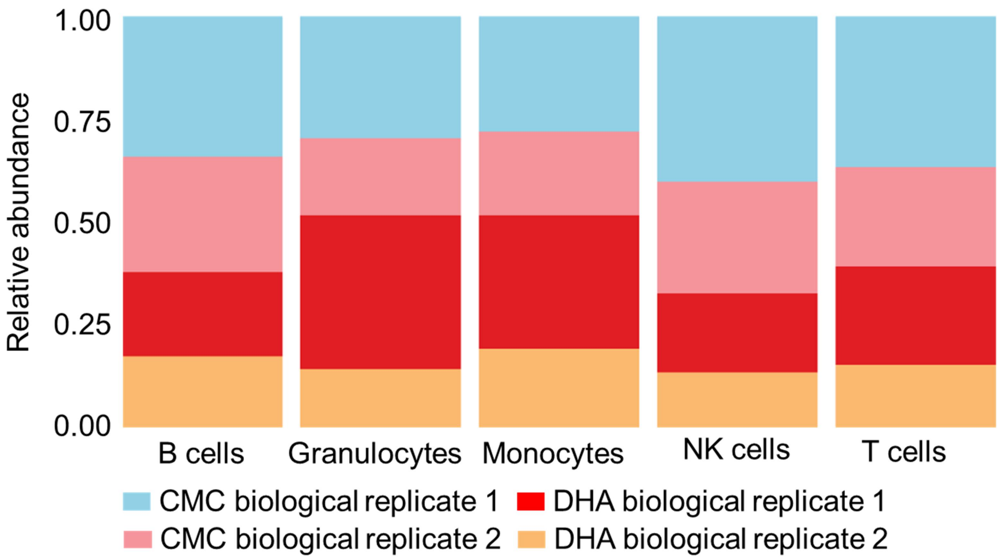

Supplementary Figure 1. The relative proportions of cell types within each cluster are examined using the stacked barplots.

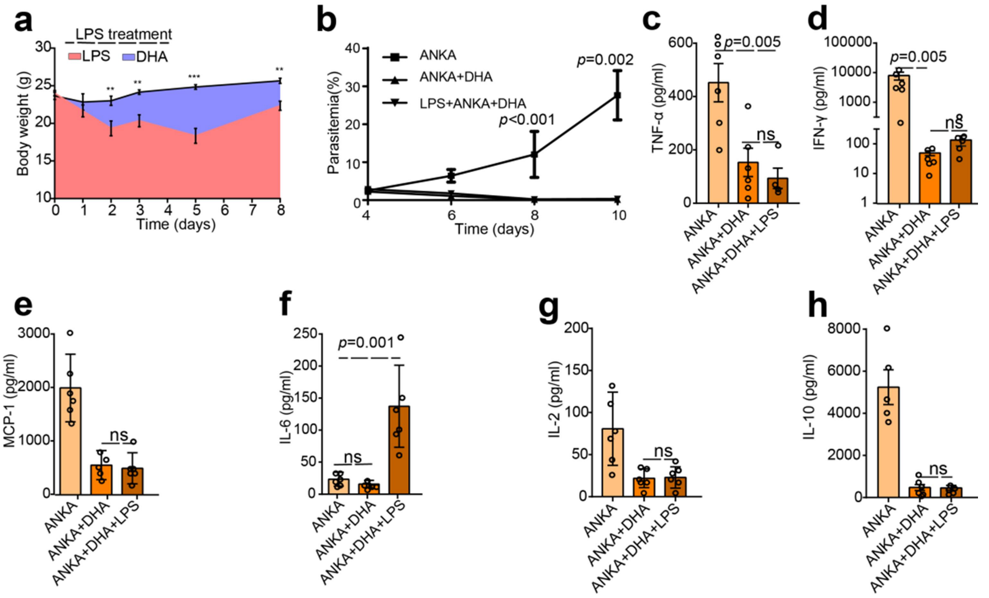

Supplementary Figure 2. (**a**) Changes in the body weights of mice treated with LPS (red line, n = 7) and mice treated with LPS plus DHA (blue line, n = 7). (**b**) Most parasites have been cleared upon DHA treatment (n = 5, Student t-test). (**c-h**) Serum abundance of IFN-γ, tumor necrosis factor α (TNFα), IL-2, MCP-1, IL-6 and IL-10 increased in *P. berghei* ANKA-infected mice compared with DHA-treated ANKA-infected or LPS- injected mice(n=4-6, Student t-test). The sera were collected at day 7 after DHA treatment and the levels of the cytokines were determined by cytometric bead array (CBA). The error bars indicate standard error.

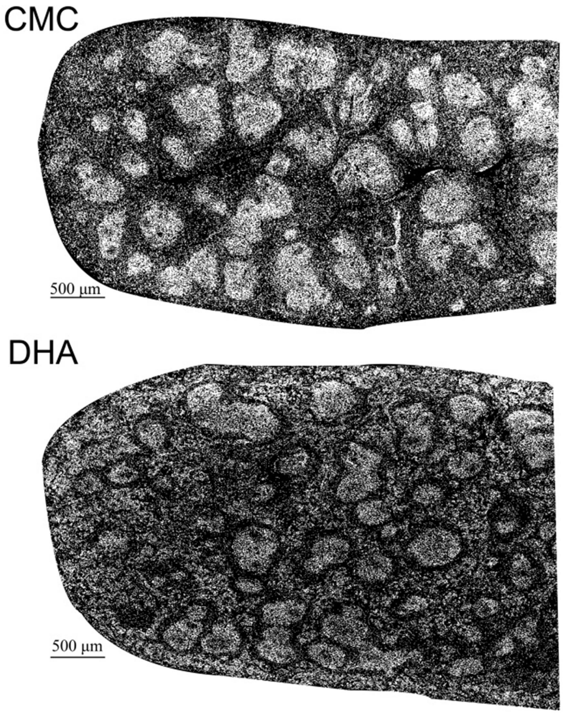

Supplementary Figure 3. Representative images of FFPE spleen sections from DHA-treated group and CMC control.

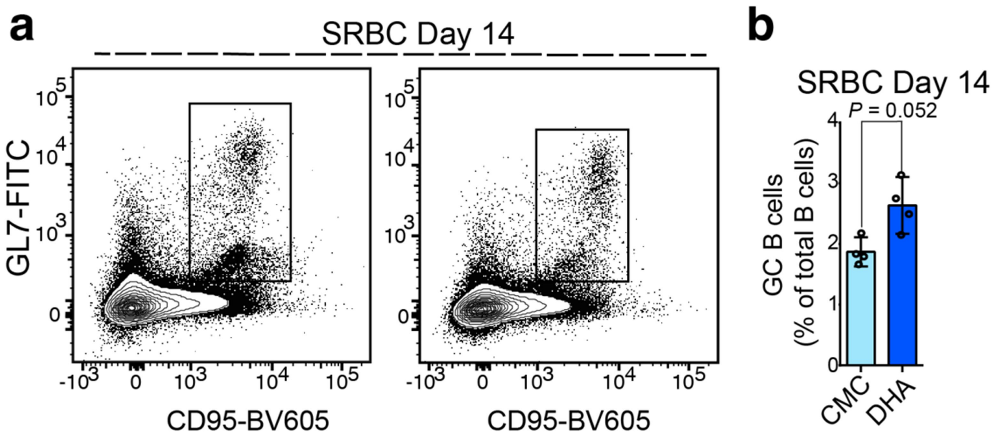

Supplementary Figure 4. (**a**) Representative flow cytometry plots of germinal center B cells ($B220^{+}CD95^{+}GL7^{+}$) post SRBC-immunization are shown. (**b**) Bar graphs represent slight and non-significant increase in percentages of germinal center B cells in DHA-treated group compared to CMC-treated group post immunized with sheep red blood cells 14 days (n=4, two-tailed Student t-test). The error bars indicate standard error.

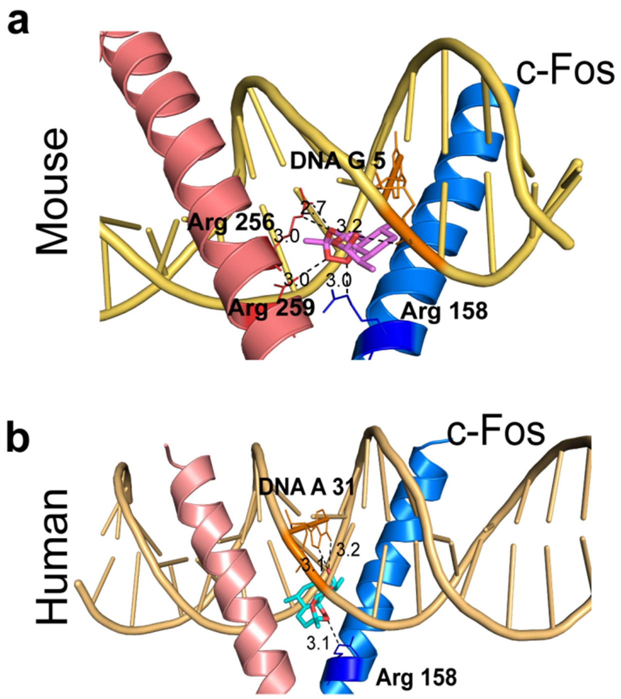

Supplementary Figure 5. Molecular docking simulations of DHA−c-Fos interactions. The docking simulation suggested that DHA could bind to both murine (**a**) and human (**b**) c-Fos close to the basic DNA binding region.

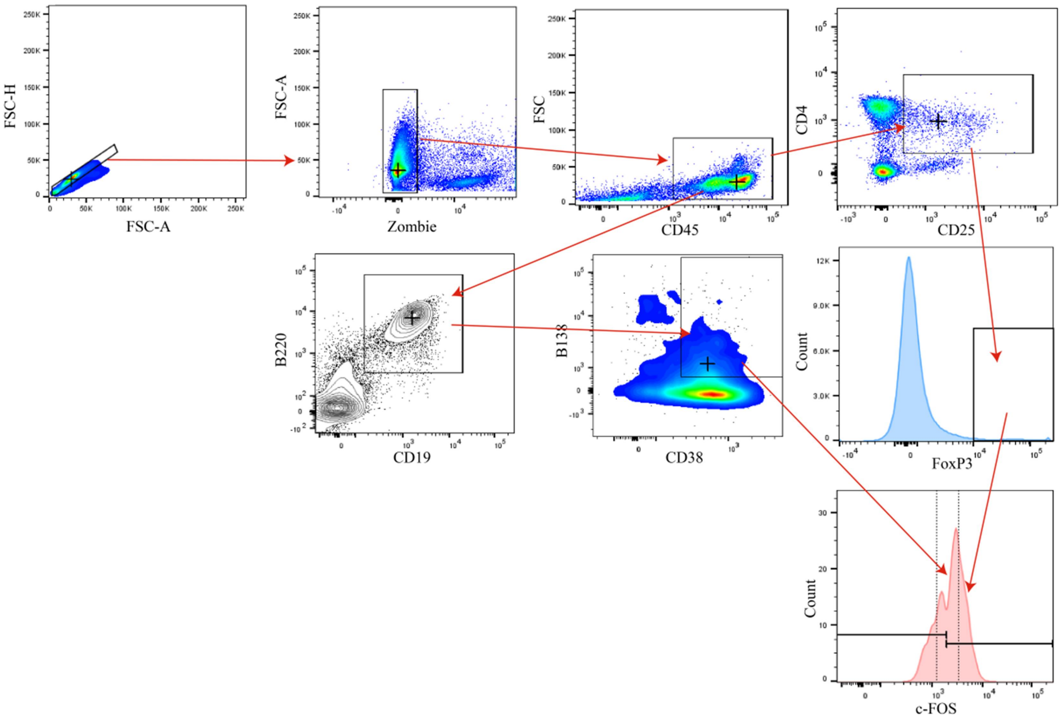

Supplementary Figure 6. Gating strategies for the detection of c-Fos. The c-Fos median fluorescence intensity (MFI) was determined by gating on Treg and plasma cells.

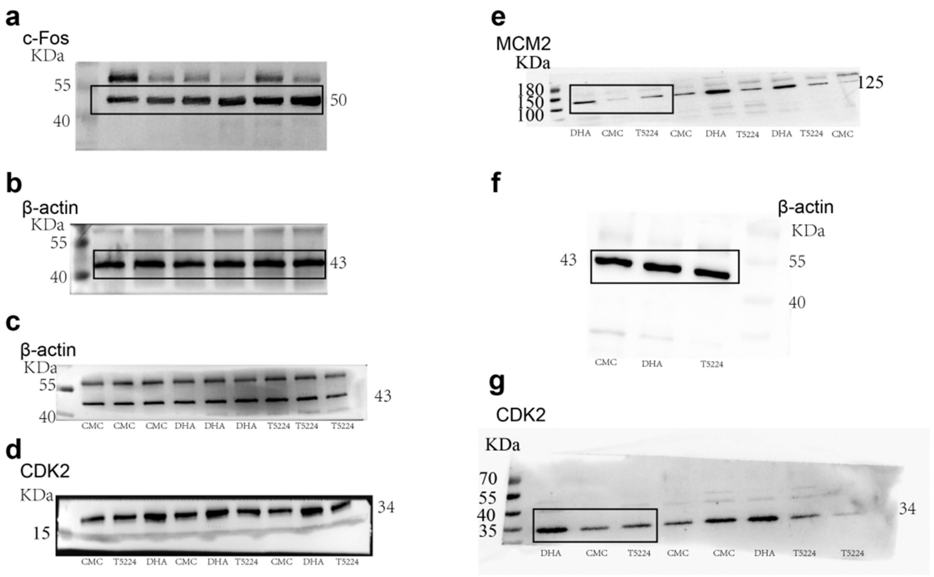

Supplementary Figure 7: Uncropped western blots correspond to Figure 6a (a and b) and Figure 6d (c-g). For c , d , the western blot experiment was repeated independently for three times with similar results.

Supplementary Data 1 Differential gene expression analysis of T_{reg} in DHA treatment compared with CMC control.

Supplementary Data 2 Differential gene expression analysis of plasma cells in DHA treatment compared with CMC control.

Supplementary Data 3 Source data of manuscript.
