# Supplementary material for: Dihydroartemisinin imposes positive and negative regulation on Treg and plasma cells via direct interaction and activation of c-Fos
Source: Commun Biol. 2023 Jan 16;6:52. doi: 10.1038/s42003-023-04454-5 (PMC9842609; doi:10.1038/s42003-023-04454-5)
Supplement: Supplementary file 3 — Description of Additional Supplementary Files [file 42003_2023_4454_MOESM3_ESM.pdf]

Description of Additional Supplementary Files

File name: Supplementary Data 1
Description: Differential gene expression analysis of Treg in DHA treatment compared with CMC control.

File name: Supplementary Data 2
Description: Differential gene expression analysis of plasma cells in DHA treatment compared with CMC control.

File name: Supplementary Data 3
Description: Source data of manuscript.
